# Supplementary figures and images for: The modified activity of prolyl 4 hydroxylases reveals the effect of arabinogalactan proteins on changes in the cell wall during the tomato ripening process
Source: Front Plant Sci. 2024 Mar 20;15:1365490. doi: 10.3389/fpls.2024.1365490 (PMC10987753; doi:10.3389/fpls.2024.1365490)

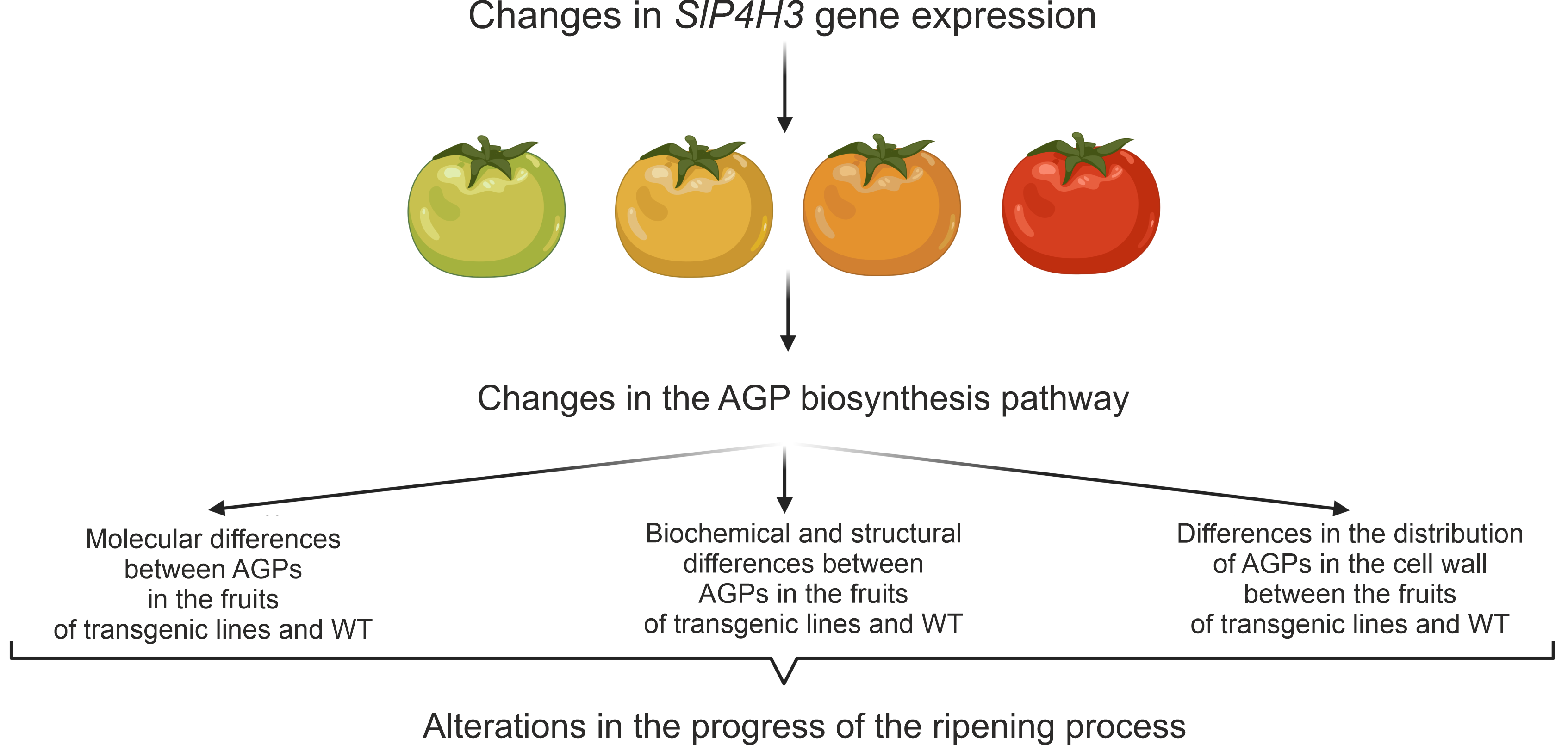

Supplement: Supplementary file 1 [file Image_1.tif]

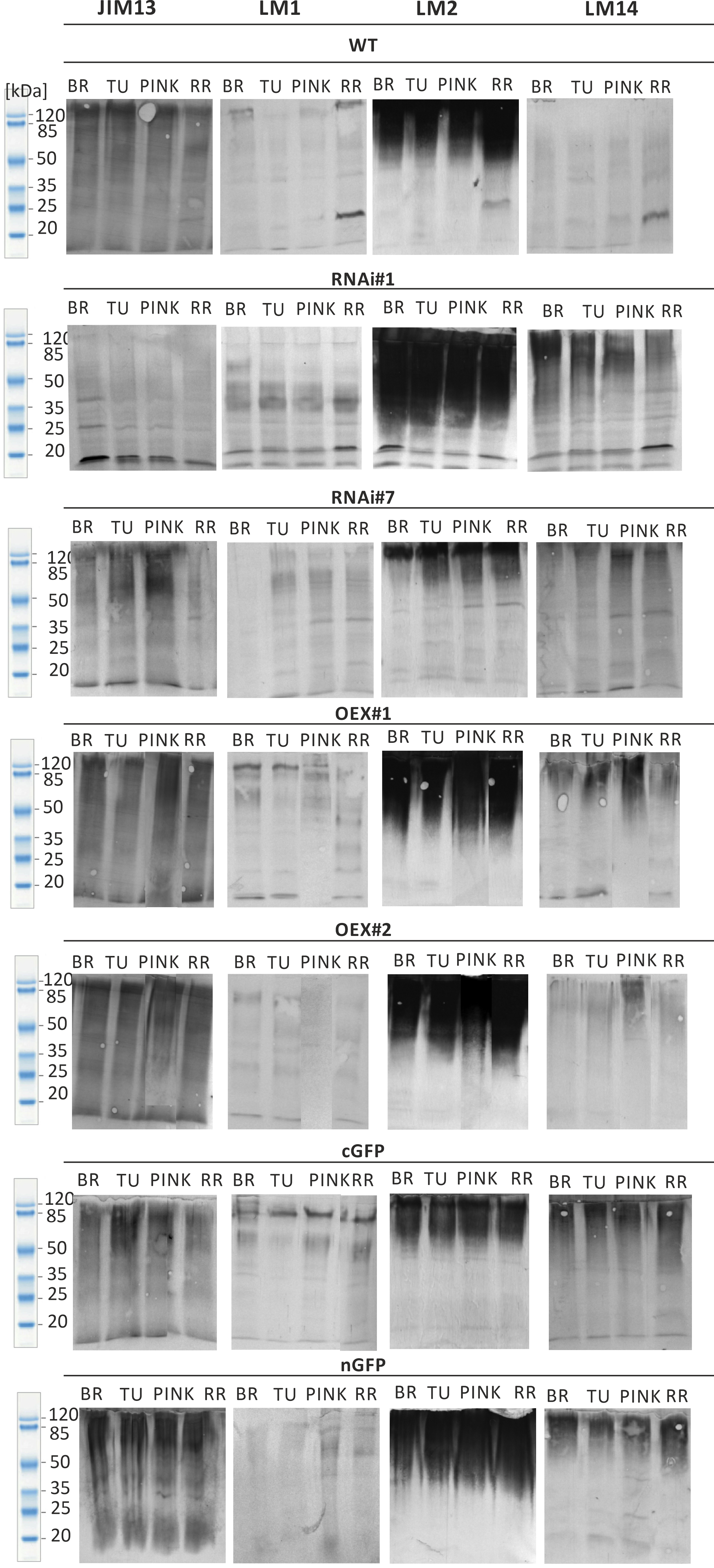

Supplement: Supplementary file 2 [file Image_2.tif]

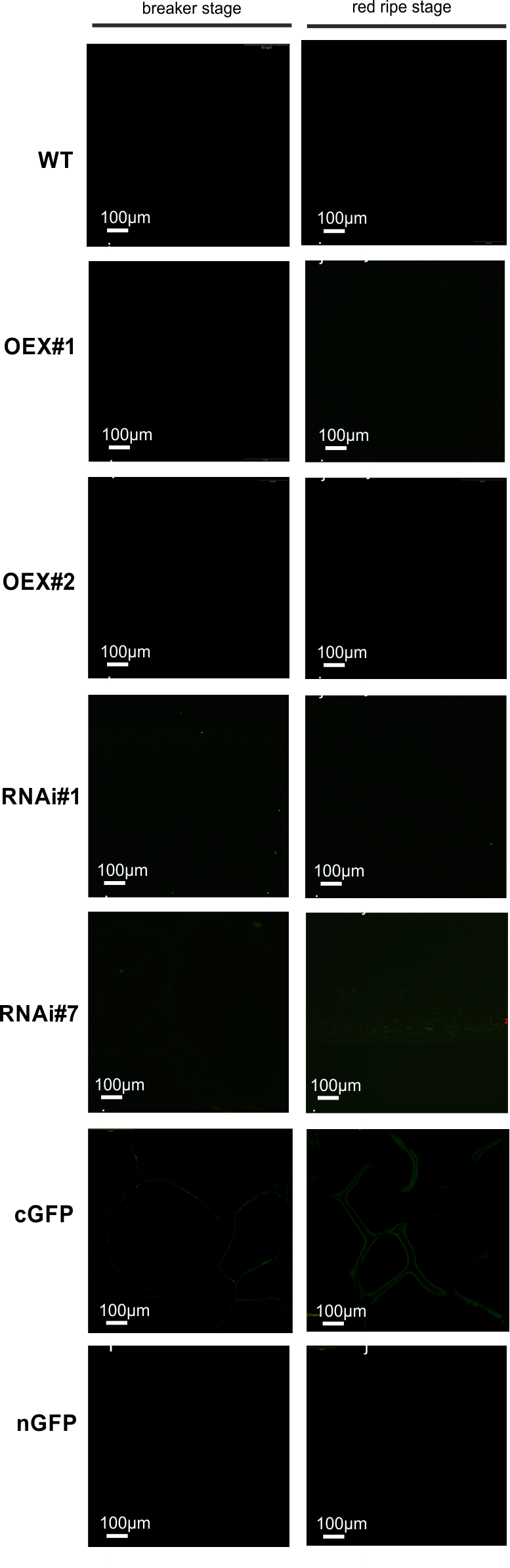

Supplement: Supplementary file 3 [file Image_3.tif]
